# Fortifying essential medicines supply chains in Sub-Saharan Africa – the curious cases of Kenya and Uganda

**DOI:** 10.1080/16549716.2026.2718931

**Published:** 2026-08-24

**Authors:** Maabo Kludze, Mustafa Hussein

**Affiliations:** Center for Immigrant, Refugee, and Global Health, Department of Health Policy and Management, Graduate School of Public Health, The City University of New York (CUNY), New York, NY, USA

**Keywords:** Universal health coverage, essential medicines, supply chains, national medicines policy, Sub-Saharan Africa

## Abstract

Universal Health Coverage (UHC) is the cornerstone of global health policy. Sustainable Development Goal 3.8 ensures access to safe, effective, quality and affordable medicines and vaccines. Sub-Saharan African (SSA) countries continue to face persistent challenges in achieving and maintaining essential medicines supply. National Medicines Policies (NMPs) that strengthen access to essential medicines are critical for UHC. In this scoping review, we take stock of the literature on essential medicines supply chains in SSA and analyze the unique, related provisions in the Kenyan and Ugandan NMPs, countries whose national essential medicines lists (EMLs) align with the WHO’s Model List and have adopted reforms specific to medicines supply chains. Comprehensive hand and electronic searches were conducted on the Kenyan and Ugandan government websites to identify recent national medicines policies and laws. PubMed and Google Scholar searches also retrieved relevant research studies. Our review highlights three key features of Kenya’s and Uganda’s NMPs: 1) development of evidence-based national EMLs, aligned with the WHO’s Model List, to guide essential medicines selection, procurement, and resource allocation; 2) devolution of central government authority for essential medicines to local counties; and 3) support and investment in domestic pharmaceutical manufacturing to minimize reliance on essential medicines imports and increase product availability. We discuss how these distinct policies can serve as models for other resource-constrained countries to build resilient, sustainable supply chains, strengthen access to essential medicines, and move closer to UHC.

## Paper Context

- **Main findings:** Kenya and Uganda provide instructive cases for strengthening supply chains of essential medicines within their National Medicines Policies, informed by the development of sound national Essential Medicines Lists and enabled by local, decentralized procurement strategies and incentives for domestic pharmaceutical manufacturing.
- **Added knowledge:** Despite a large literature on essential medicines and universal health coverage, related supply chain challenges have received less attention. We review the literature on essential medicines supply chains in low- and middle-income countries, focusing on Sub-Saharan Africa, and identify and elaborate relevant lessons from two countries whose policies uniquely address these challenges.
- **Global health impact for policy and action:** Wider, tailored adoption of similar policy ideas as in Kenya and Uganda can help make essential medicines supply chains more resilient, equitable, and sustainable.

## Background

The constitution of the World Health Organization (WHO) enshrines as a founding principle the right to health and the opportunity to achieve the highest attainable standard of health across all stages of life [1]. Universal Health Coverage (UHC) provides a global umbrella to guide the equitable financing and delivery of health services across countries, especially in the developing world [2,3]. The Sustainable Development Goal (SDG) 3.8 explicitly lists achieving UHC through securing ‘access to safe, effective, quality, and affordable essential medicines and vaccines for all’ as one fundamental aspect of UHC [4]. Yet, approximately 2 billion people, most of whom are in low- and middle-income countries (LMICs), cannot secure reliable access to medicines, particularly those deemed essential for their well-being [5–7]. The WHO defines essential medicines as ‘those that satisfy a population’s priority healthcare needs. They are intended to be available in functioning health systems at all times, in appropriate dosage forms, of assured quality, and at prices individuals and the community can afford’ [8]. Essential medicines are critical in improving health outcomes, and policies addressing their access remain a global priority. In fact, a stepping stone on the path to UHC involves identifying and constructing the essential benefits package, of which essential medicines are a critical component [9,10].

The WHO introduced the first Model List of Essential Medicines (MLEM) in 1977 to address significant public health concerns [11]. The MLEM is a reference for all national essential medicines lists (EMLs) that can be adapted to reflect new healthcare practices, adoption of technology, national disease trends, and innovations in drug therapy [11]. As such, differences exist between national EMLs and the WHO’s MLEM [12,13]. Piggott et al. showed that many countries struggle to fully align their national EMLs with the WHO MLEM [14]. Alignment was measured by an F1 score, with 1 indicating perfect alignment. The overall global median F1 score was 0.59, indicating moderate alignment and highlighting opportunities for improvement [14]. In a related study, Persaud et al. concluded that the differences between national EMLs and the WHO MLEM in 137 countries were due to differences in geographic region, population, or healthcare investments [13]. These findings also underscore the wide-ranging capabilities and oft-constrained resources needed to adopt and optimize EMLs [15].

Beyond building country EMLs and alignment with the WHO MLEM, the procurement of essential medicines through sustainable, resilient supply chains has received comparatively less attention, particularly in sub-Saharan Africa (SSA). Although SSA countries have committed to achieving sustainable, efficient, and reliable medicines supply chains, many continue to face persistent challenges. This scoping review focuses on two SSA nations, Kenya and Uganda, whose national EMLs closely align with the WHO MLEM (75% and 68%, respectively) [14,16] and whose essential medicines policies have made specific supply-chain reforms and steady progress toward UHC.

Kenya and Uganda are thus uniquely instructive, more so than regional peers. For example, Tanzania’s 2023 *Universal Health Insurance Act* mandates enrollment and funds high-cost non-communicable disease treatments (e.g. cancer) but does not address essential medicines [17]. Malawi’s *Health Sector Strategic Plan III (2023–2030)* emphasizes integrated primary care but lacks explicit guidance on improving essential medicines supply chains [18]. Botswana’s 2011 *Essential Health Service Packages for Botswana* and the *National Health Policy: Towards a Healthier Botswana* [19–21] focuses on equitable access, yet it too does not include specific direction on essential medicines. Rwanda’s *Health Sector Strategic Plan (HSSP) V (2024/25–2028/29)* suggests significant progress was made with essential medicines availability between 2020 and 2023, with little detail on strategies [22]. Conversely, the Zimbabwe *National Health Strategy (NHS) 2021–2025* does address supply chain strategies and its UHC Service Coverage Index, a marker for attainment of SDG 3.8.1, has reached 59 in 2023 [23,24]. However, evidence suggests that Zimbabwe’s pharmaceutical supply chain is chronically underfinanced, with weak governance and accountability and a significant reliance on external donor financing [25].

The UHC Service Coverage Index evaluates the service capacity and access components in SDG 3.8.1, and ranges from <20 (very low coverage) to ≥80 (high coverage) [24]. Kenya had a score of 57 in 2023, reflecting a 29-point increase from 2000, and Uganda had a score of 54, a 30-point increase from 2000. Both countries surpass the African regional average of 47 within the same time frame [24,26], reflecting steady progress toward the UHC [24,27]. While various factors likely played a role in this improvement, the government’s inclusion of essential medicines into multiple policies has likely contributed.

Kenya and Uganda further distinguish themselves with accelerated coverage of essential medicines in the last 25 years, including rising scores on medicines service capacity and the creation of expanded robust EMLs. Additionally, these countries have undergone recent UHC policy reforms for improving their essential medicines supply chains [28]. These reforms position Kenya and Uganda as regional innovators in blending structural UHC changes with targeted medicines access strategies, contrasting with peers whose reforms remain incremental or less explicit in addressing essential medicines.

The aim of this scoping review is to systematically identify and analyze the national policies intended to strengthen the supply and availability of essential medicines in Kenya and Uganda, as models for SSA countries and similar resource-constrained settings. We first review the literature on essential medicines supply chain systems in SSA countries and the core challenges they face. A focused description of the supply management components of the National Medicines Policy (NMP) that address essential medicines then follows. Finally, the NMPs of Kenya and Uganda are used as case studies to demonstrate how key supply chain-related provisions can accelerate progress toward better access to essential medicines on the path to UHC.

## Methods

### Study framework

This review followed the framework for scoping reviews under the Preferred Reporting Items for Systematic Reviews and Scoping Reviews (PRISMA-ScR) guidelines [29,30]. Both authors collaboratively developed the protocol and reviewed the studies internally. The PRISMA-ScR approach is well-suited for this study as it allows for exploring the multifaceted literature and for using specific cases as illustrative examples to identify best practices and inform future work [29].

### Eligibility criteria

Table 1 lists the inclusion criteria and rationale guiding our publication search and review process. The literature review included (1) English-language publications that analyzed (2) supply chain policies applicable to essential medicines in sub-Saharan Africa, especially (3) Kenya and Uganda, including (4) published peer-reviewed studies within a 10-year period (2015–2025).

**Table 1.** Criteria and rationale for inclusion of studies in this review.

Study Inclusion Criteria	Rationale
Analyze essential medicines supply chain policies in Sub-Saharan Africa	To uncover types of policies that specifically address supply chains for essential medicines in sub-Saharan country contexts
Address supply chains in the national medicines policies in Kenya and Uganda	To identify and review policies in Kenya and Uganda as instructive case studies for strengthening essential medicines supply chains in peer countries
Published between 2015–2025	This time frame aligns with accelerated coverage and supply chain reforms for essential medicines in sub-Saharan Africa and was selected to capture relevant, current and contemporary models and policies in supply chain and medicines delivery in the region.

### Information sources

Peer-reviewed, original research articles and grey literature from the Kenyan and Ugandan government websites were all considered; however, only publications available in full text were included. Abstracts, editorials, perspectives, commentaries, opinions, PhD theses, and clinical reviews were excluded. Peer-reviewed and original research articles are needed to focus on essential medicines supply chains in SSA. Publications were excluded if they did not address supply chain provisions or if they discussed the use of specific essential medicines without linking to supply chain structures or policies. Efforts were taken to ensure that national policies, strategic plans, and documents were current and active.

### Search strategy

A modified version of Arksey and O’Malley’s scoping review methodology [31] guided our approach. Table 2 outlines our search strategy. From April through August 2024, we used a combination of hand and electronic database searches to identify relevant publications in each country [32]. An additional publication search was conducted in October 2025 for updates, which identified two additional articles published in 2025, which did not meet our eligibility criteria.

**Table 2.** Search strategy phases and keywords.

	Concept #1	Concept #2	Concept #3	Concept #4
Initial database search	essential medicines	supply chain	sub-Saharan Africa	Kenya and Uganda
Targeted grey literature search	Kenya	NMPs, laws, national guidelines, strategic action plans, and health policy	essential medicines	supply chains
	Uganda			
Supplementary search	Kenya	domestic manufacturing	Kenya Medical Supplies Authority, Kenya Health Policy 2014–2030, the Kenya Universal Health Coverage Policy 2020–2030	Kenya Constitution, Kenya Essential Medicines List
	Uganda	domestic manufacturing	Vital, Essential, and Necessary classification system	Buy Uganda, Build Uganda policy

Searches for eligible publications were conducted in three sequential phases with each building on prior searches to refine the scope. The initial broad search in PubMed and Google Scholar using ‘essential medicines AND supply chain AND Sub-Saharan Africa’ provided regional context and helped identify common frameworks, challenges, and themes. Reference lists from eligible studies were hand-searched to identify further relevant articles. Subsequent screening and review of full-text documents identified Kenya and Uganda as countries with EMLs highly aligned with WHO’s MELM and with effective national policies on medicines supply chains. As a result, these countries were chosen for in-depth analysis.

**Figure 1.**
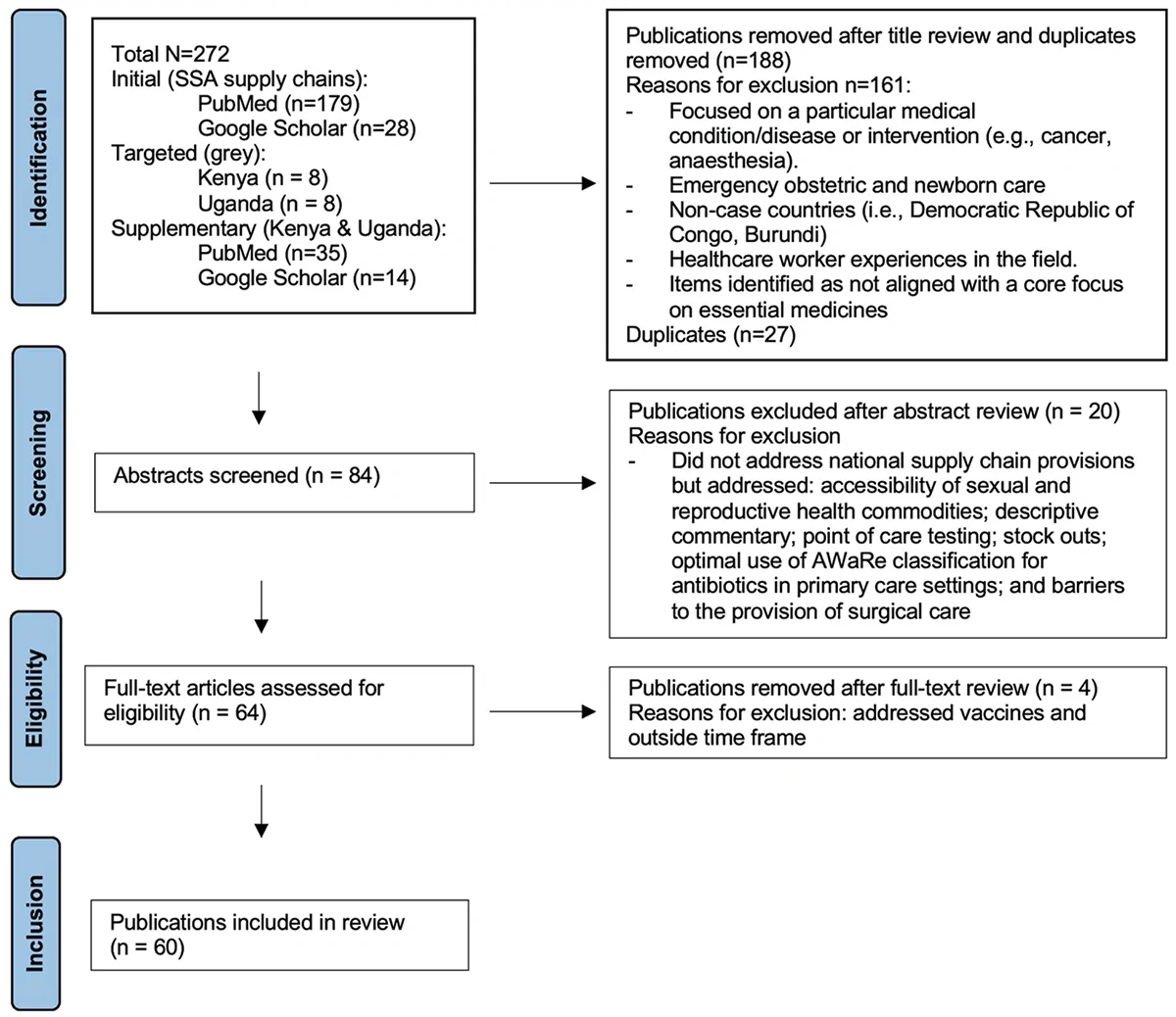
Flow diagram of searches and inclusion process for publications on national policies for essential medicines supply chains in Sub-Saharan Africa, 2015–2025.

The secondary, more focused search involved searches on the Kenyan and Ugandan government websites and online databases. These searches enabled identifying the grey literature on recent NMPs, laws, national guidelines, strategic action plans, and health policy documents related to essential medicines supply chains (Figure 1). The Laws of Kenya and the Guidelines, Standards & Polices Portal webpages were searched using the terms ‘essential medicines’ and ‘supply chains.’ The same process was used on the Ugandan Ministry of Health Knowledge Management Portal. This phase resulted in sixteen policy-related documents, eight from each country.

Finally, the third phase employed a targeted search in PubMed, informed by findings from the grey literature, to delve deeper into significant supply chain provisions specific to Kenya (such as domestic manufacturing initiatives) and Uganda (such as the Buy Uganda, Build Uganda policy). Searches on PubMed used these country-specific keywords to identify publications focused on policy and implementation outcomes, to allow for more in-depth understanding of the impacts of these strategies.

Retrieved studies were selected, charted, and collated as outlined in Figure 1, following the relevant methodological advice [31]. The co-authors reviewed the list of eligible articles after title, abstract, and full-text review and approved the final list of included articles. The complete manuscripts of the remaining articles were then assessed against the eligibility criteria.

## Results

A total of 272 articles were retrieved across search phases, and 208 articles were excluded, leaving 64 articles for full-text review (see Figure 1). Finally, a total of 44 peer-reviewed articles and 16 national documents were included in this scoping review. The filled PRISMA-ScR checklist can be found in Supplemental Table 1, with all 60 included studies summarized in Supplemental Table 2.

Table 3 outlines the key similarities and differences in supply chain provisions in Kenya and Uganda’s essential medicines policies, elaborated next.

**Table 3.** Key features of essential medicines supply chain policies in Kenya and Uganda.

	Kenya	Uganda
Number of medicines on National Essential Medicines List	696	784
National EML alignment with the WHO EML	75%	68%
UHC Service Coverage Index, 2023	57	54
Policies and provisions governing essential medicines	Kenya Essential Medicines List, 2023 Kenya Health Policy 2014–2030 Kenya Universal Health Coverage Policy 2020–2030 Decentralization of health services Kenyan Constitution The Health Act, 2017	The Essential Medicines and Health Supplies List of Uganda, 2023 Vital, Essential, and Necessary classification system Buy Uganda, Build Uganda policy establishes legislation for domestic manufacturing of medicines and establishes a regulatory agency and a public system to manage medicine supply Decentralization of health services
Distribution of essential medicines	Dual-sector methodology to distribute medicines: Public: Kenya Medical Supplies Authority, a state-run corporation Private: Counties use Mission for Essential Drugs and Supplies or private distributors; for-profit or not-for profit donations)	Dual-sector methodology to distribute medicines: Public: National Medical Store and the Joint Medical Store Private: For-profit or not-for profit donations
Domestic manufacturing	The Health Act, 2017 Ministries of Health and developed the Kenya Pharmaceutical Sector Development Strategy, 2012	Buy Uganda, Build Uganda policy
Domestic manufacturing incentives and support	The KEML contains language on expedited drug registration for local manufacturers, and incentives for local production that primarily target products listed on the KEML. Roadmap that guides pharmaceutical industry to achieve the WHO Good Manufacturing Practice Standards	Legislation provides incentives for domestic manufacturers through tax benefits and subsidies Increase in import verification fees
Devolution model	County-level autonomy	District-level management

EML: Essential Medicines List, KEML: Kenya Essential Medicines List, WHO: World Health Organization, UHC: Universal Health Coverage.

### Essential medicines supply chains in Sub-Saharan Africa

In the past decade, the promise of supply chains for strengthening health systems has been globally acknowledged [33]. Supply chain management is a well-studied discipline that seeks to optimize the intricate ecosystem of suppliers, information technology, and various stakeholders, and the flow of information, products, and finances throughout the system. The medicines supply chain process begins with acquiring raw materials, then manufacturing, and packaging of the finished product. The finished product then enters the management cycle on the demand side, which involves the following core functions: rational selection, quantification and forecasting, procurement, storage, and distribution [34]. Seamless delivery of medicines from the manufacturer to the patient requires commitment and coordination among stakeholders throughout the supply chain [33,35]. In many SSA countries, medicines are distributed through three sectors: the public or government, the private not-for-profit non-governmental organizations, and the private commercial for-profit sector. Distribution models vary across SSA countries, with some utilizing central medical stores or regional/district depots as primary distribution hubs, and then drug shops, medicines retailers, or pharmacies which provide the majority of essential medicines and basic healthcare services to patients using different models [36–39]. Targeted, long-term initiatives such as Project Last Mile partnered with the private sector to optimize delivery systems, with strategies borrowed from Coca-Cola (e.g. investing in local relationships), leading to stronger distribution channels [40].

The presence of multiple independent medicines supply chains in SSA countries introduces complexities and overlapping activities; however, it can also be viewed as an opportunity. The overlapping supply chains can protect against shortages by increasing the likelihood of access for patients and introducing competition across pharmaceutical manufacturers and could possibly lower prices and improve quality. On the other hand, having multiple chains makes it difficult to centrally plan, forecast, and monitor the supply of essential medicines on the government end, and to introduce uniform reforms. The various weak points across chains make it difficult to promptly manage issues as they arise. There are also significant challenges in efficiently managing medication orders, storage, distribution, and demand forecasting. Lack of a robust, uniform information management system that provides real-time data to assess stock levels, predict demand, and communicate this demand to stakeholders further complicates the process [41–47]. Together, these challenges weaken the supply chain system and undermine the health system’s ability to reach SDG 3.8 goals.

### National medicines policies and essential medicines

A NMP represents a government’s commitment and plan of action to improve its pharmaceutical sector. NMPs have evolved to play a critical role in addressing access to essential medicines over the last several decades. From the 1970s to the 1990s, essential medicines policies focused on integrating the concept of essential medicines as a component of primary healthcare and creation of national EMLs to maintain [48–50]. Financial and political investments drove the second phase of policy development toward expanding access to medicines, especially to address the growing public health concerns of Acquired Immune Deficiency Syndrome (AIDS), tuberculosis, and malaria in SSA, from the 1990s to the 2010s [49]. In more recent years, NMPs have transitioned to align with UHC and its related SDGs by focusing on securing therapies for noncommunicable diseases and ensuring equitable access [49,51]. The supply chain management component of NMPs often addresses the implementation strategies for the selection and procuring of essential drugs based on the population’s health needs. The WHO recommends integrating the NMP into national health systems so that the government’s commitment to providing medicines is translated into reality [52].

By 2013, about 90% of SSA countries established initial iterations of EMLs and NMPs [49]. Perehudoff evaluated NMPs and UHC legislations across several countries to determine if provisions exist for essential medicines [12]. Among the 50 LMICs, 78% had national EMLs, 82% had NMPs, and 8% addressed access to medicines in their constitutions [12]. The authors found that 23.9% (17 out of 71) of NMPs and 50% (8 out of 16) of UHC legislations made strong provisions for essential medicines. For example, Ghana’s UHC framework and NMP underscore access to essential medicines as part of the fulfillment of the human right to health [53]. Ghana regularly updates its EMLs to reflect the country’s disease burden and uses cost-effectiveness criteria. Its National Drugs Policy, updated in 1999 and 2004, emphasizes local manufacturing while the 2017 NMP provides tax exemptions for local manufacturing [54].

Alignment of national EMLs with the WHO MLEM varies across countries [13,14]. In a study by Persaud et al., no country included all the 414 medicines on the 2017 WHO’s MLEM medicines in their national list [13,55]. Each country’s EML contained between 44 and 983 medicines with a median of 310 medicines. The median difference between each national list and the WHO MLEM was 296. Only two SSA countries, Ethiopia and Kenya, included more than 300 WHO MLEM drugs [13,55]. Kenya and Uganda were further standouts among SSA countries, showing high alignment with the WHO MLEM, with Kenya including 310 medicines (75%) and Uganda including 247 medicines (68%). Overall, these findings suggest ongoing gaps in securing access to essential medicines at levels consistent with SDG goals for UHC in LMICs, especially SSA countries [12,13,50,56,57].

Although national EMLs have a significant influence on procurement, prescribing, and financing systems, there is limited research evaluating how national EMLs in SSA countries inform and drive supply chain systems. For example, selecting medicines from the WHO MLEM can have significant budgetary implications in resource-constrained settings [57–60]. Peacocke et al. note that for countries to obtain the greatest value from their national EMLs, significantly more funding, personnel, and stakeholder commitment are needed [61]. These additional resources are also required for governments to align their national EMLs with the WHO MLEM and to incorporate essential medicines provisions in their broader UHC strategy.

### Provisions for essential medicines in the national medicines policies of Kenya

#### Procurement and distribution of essential medicines

Kenya, with a population of 55.1 million, is considered the regional economic and pharmaceutical center of East and Central Africa [62,63]. Access to healthcare is a fundamental human right in the Kenyan Constitution [64]. The government plays a central role in regulating national health policy, providing technical support, and managing health institutions and programs. Like many SSA countries, Kenya employs a dual-sector methodology to distribute medicines, utilizing both public and private systems [65]. In the public sector, the state-run corporation Kenya Medical Supplies Authority (KEMSA) is the primary body responsible for procuring drugs [28]. While KEMSA serves as the main distributor for most public health facilities, counties often use alternate sources such as the Mission for Essential Drugs and Supplies (MEDS) or private for-profit distributors as donations [66]. Alternate pathways that circumvent KEMSA bypass the Ministry of Health’s direct oversight and do not require pre-market approval from the Kenyan Pharmacy and Poisons Board. Consequently, medicines entering through private channels, often as donations, circumvent the government’s established approval procedures [62,66].

Medicine procurement through KEMSA performs lower than the private sector due to limited availability, particularly essential medicines for non-communicable diseases. Data from 2018 to 2019 revealed that the mean availability of essential medicines on hand was 44% in public facilities compared to 72.4% in the private sector [67]. A 2020 policy brief by the Kenyan MOH further highlighted this disparity, noting that dispensaries and health centers had lower stocks of essential medicines (33% and 50%, respectively) compared to 70% at hospitals [67]. Overall lack of access often results from both public sector stockouts due to insufficient funding and lower affordability in the private sector where medicines are more available but at higher prices and out-of-pocket costs [51,68–71]. These dual challenges of availability and affordability underscore the need for robust policies to strengthen procurement systems, address stockouts, and regulate medicine pricing to ensure equitable access for all.

Over the last 20 years, the Kenyan government has introduced various policies and strategies to improve the availability of medicines within the supply chain. These efforts have significantly enhanced childhood survival rates, improved maternal care outcomes, and increased access to health insurance. The following paragraphs will review Kenya’s essential medicines provisions across policies and their influence on access, including the Kenya Essential Medicines List (KEML), the Kenya Health Policy 2014–2030, the Kenya Universal Health Coverage Policy 2020–2030, and the impact of the decentralization of health services as outlined in the Kenyan Constitution [28,64,72].

#### Kenya’s essential medicines list

Kenya’s updated 2023 KEML includes 35 therapeutic categories and 696 essential medicines [28]. The 2023 update to the KEML reflects the evolving healthcare needs of the population and serves as a critical tool to promote rational use of medicines and to inform essential medicines and UHC policies [28]. By focusing on a prioritized, needs-based list of essential items, the KEML seeks to enhance the efficiency and effectiveness of medicines supply systems, streamlining operations across all levels. The KEML is a foundation for a comprehensive regulatory framework that governs all aspects of medication management, from importation and exportation to local production, registration, procurement, distribution, and dispensing. The KEML establishes a framework for determining medicines procurement needs across all healthcare sectors. Additionally, the KEML provides a standardized process that county governments may use to procure medicines and estimate budgets across various healthcare system levels while maintaining alignment with national objectives. For domestic manufacturing, the 2023 KEML contains strategies for expediting registration procedures and offering incentives to promote local pharmaceutical production of medicines included in the KEML.

#### Provisions for essential medicines under Kenya’s health policy reforms

In addition to the 2023 KEML, Kenya enacted two important policies: the Kenya Universal Health Coverage Policy 2020–2030 and the Kenya Health Policy 2014–2030 that focus on enhancing access to health products and technologies, including essential medicines and vaccines [73,74]. The Kenya Universal Health Coverage Policy 2020–2030 calls explicitly for improvements in the availability, accessibility, quality, and pricing of medicines, through the National Hospital Insurance Fund (NHIF) [75]. One study noted that although reforms to the NHIF scheme provided comprehensive benefit packages on paper, members often had limited access to medicines due to frequent unavailability at contracted healthcare facilities [75]. This policy also addresses the need for adequate forecasting and quantification every 2 years at the facility and county levels as according to national guidelines.

On the other hand, the Kenya Health Policy 2014–2030 leverages public and private investments to establish effective and reliable procurement processes [74]. Within this policy is the Health Products and Technologies Supply Chain Strategy 2020–2025, which seeks to improve coordination and inform decision-making and investments across health products and technologies [76]. These investments build a reliable and sustainable supply chain system by creating strategic reserves. Under this policy, the national government is expected to purchase and maintain adequate stocks of health products and essential medicines that fall into three main categories: *Strategic* (including vaccines and drugs for TB, HIV/AIDS, and epidemics), *Special and Expensive* (such as cancer drugs and immunosuppressives), and *Essential or Basic* products [74]. This categorization aims to streamline resource allocation and create a clear process for building a national stock reserve [77]. Recognizing the potential for local production, in 2012 the ministries of industrialization and health created the Kenya Pharmaceutical Sector Development Strategy and a roadmap to guide domestic manufacturers in achieving the WHO good manufacturing practices; this was a strategic step in the implementation of Kenya’s NMP [74].

#### The role of Kenya’s devolved system

Besides these two policies and the 2023 KEML, Kenya’s constitution leverages the devolved government system as the foundation for all efforts, ensuring that resources and decision-making are more closely aligned with local needs and priorities. In 2010, the Constitution of Kenya introduced a devolved system whereby the country’s 47 local counties were granted authority to manage the healthcare systems on a local level [72,78]. Under this arrangement, local governments possess the autonomy to determine and allocate healthcare funding and commodities that meet county needs but still align with the national healthcare goals. Unlike other devolved systems, all health service delivery functions, including the procurement of essential medicines and medical supplies, are also assigned to county governments, rather than central governments [78]. This type of management directly reflects the local supply and demand, creating a more targeted and efficient procurement process [78]. This was a significant departure from the longstanding centralized governance viewed as an inefficient, unresponsive, and distributing health services in an unbalanced manner [78,79]. This decentralized system better positions county governments to respond quickly to changes in disease prevalence, prevent delays in providing essential medicines, and improve distribution networks to meet the needs of geographically remote people [80]. Local counties are better positioned to make those decisions and allow for a more effective process of determining supply with local demand. District-level facilities are encouraged to utilize national guidelines for redistribution strategy for essential medicines to prevent and manage expired medicines due to over and under stocking. Other models, like the Revolving Fund Pharmacy, reinvest medicines sales revenue to procure essential medicines which supports sustainability and capacity of the supply chain [81].

### Essential medicines under Uganda’s devolved system and NMP

In 2022, Uganda had an estimated population of 47.2 million people, projected to increase by 85% by 2050, placing a significant burden on the healthcare system [82]. Healthcare services are funded by the government, private interests, and investment partners but are, like Kenya, delivered through a devolved system [83]. Uganda’s devolved government system was created in the 1990s through shared responsibility whereby the MOH establishes policies and strategic direction while district governments manage service delivery. The MOH plays a coordinating role in the medicines supply chain by overseeing the implementation of pharmaceutical policy, setting national pharmaceutical product requirements, promoting the rational use of medicines and streamlining supply chain management systems. The government funds two supply chains that account for 95% of all medical products in Uganda – the National Medical Store (NMS) and the Joint Medical Store (JMS) [84–86]. The NMS was established as a statutory corporation in 1993 to play the role of the central medicines service to procure, warehouse, and distribute pharmaceutical products to all public health facilities.

This central medicine service is supported by the NMP, which was introduced in 2015 to address ongoing challenges in medicines delivery [87]. Uganda’s NMP emphasizes three key principles: quality of care, equity, and the efficient use of available resources. Its focus includes advancing UHC, increasing funding for medicines, strengthening health partnerships and collaborations, and enhancing the capacity of the MOH’s and district health departments [88].

#### The role of Uganda’s essential medicines list

The Essential Medicines and Health Supplies List of Uganda (EMHSLU) and the Vital, Essential, and Necessary (VEN) classification system guide the selection of essential medicines for population health needs [83,87,89,90]. Both classification schemes categorize medicines based on their significance and impact on public health. The EMHSLU aligns with Uganda’s NMP and provides a standardized framework for selecting and procuring medicines across public and private sectors. This integration ensures that medicines are selected based on evidence, population health needs, and cost-effectiveness. The VEN system similarly aims to optimize resources and prioritize medicines into three categories. *Vital* medicines (V) are essential for diagnosing and treating life-threatening conditions or serving as first-line therapies. Their uninterrupted availability is critical to preventing significant public health risks. *Essential* medicines (E) are used for common illnesses, such as migraines, or as second-line therapies for conditions of lower criticality. Finally, the *Necessary* medicines (N) treat self-limiting minor conditions such as diarrhea, which pose minimal health risks to the population. This VEN classification guides health facilities in procuring medicines to mitigate the risk of harm, thus optimizing resource allocation. A national unit dedicated to medicines procurement, demand forecasting, and overall supply chain coordination is also mentioned in the NMP [83,87,89,90].

#### Uganda’s local pharmaceutical manufacturing strategy

Uganda’s NMP includes a unique national strategy aimed at ensuring a reliable supply of essential medicines. This strategy focuses on reducing dependence on imports by implementing a national policy that encourages the development of local pharmaceutical manufacturing [87]. The NMP also outlines a plan to strengthen the pharmaceutical manufacturing sector by developing legislation and establishing a regulatory agency for pharmaceuticals, and implementing a public system to manage medicines supply [89]. Legislation provides incentives for domestic manufacturers through tax benefits and subsidies [91]. Domestically manufactured products are promoted for procurement by national and international agencies. Uganda offers the same incentives to other East African Community countries, which fosters regional cooperation and growth [87]. Uganda’s national strategy was operationalized in 2017 as the Buy Uganda, Build Uganda (BUBU) policy. The BUBU increased import verification fees on 35 essential medicines locally manufactured from 2% to 12% [92,93]. The BUBU also imposes a differential tax on imported products as a deterrent [89,92]. The BUBU increased local production capacity by 8.2% from 2017 to 2020 and the demand for locally produced medicines also increased [92]. The number of domestic medicines manufacturers also grew from 11 to 29 to 42 in 2009, 2021, and 2022, respectively [89,94,95]. However, in 2023, only 10% of the locally manufactured medicines were essential [96]. Since the program is in its infancy, imported products remain more widely available than those locally produced [97].

## Discussion

This scoping review identifies three key policies implemented by Kenya and Uganda that could serve as models for strengthening supply chains and improving access to essential medicines under the UHC framework: 1) adoption and tailoring of the WHO MLEM into national EMLs to guide selection and procurement practices; 2) devolution of government authority for healthcare services; and 3) policies supporting investment in domestic pharmaceutical manufacturing to help minimize reliance on essential medicines imports and increase product availability.

### Alignment with the WHO MLEM

Aligning national EMLs with the WHO MLEM can be part of a broader set of initiatives to strengthen pharmaceutical procurement in SSA countries, leading to greater access. Kenya and Uganda demonstrate high alignment with the WHO MLEM, which promotes evidence-based rational use, reduces waste, and optimizes pharmaceutical resource allocation. National EMLs can be particularly effective when product selection and purchasing are linked to financing and procurement systems. This strategy has been implemented in various forms in non-SSA countries as well. The essential medicines provisions in South Africa and China shape pricing and procurement by integrating EMLs into funding and purchasing systems [98,99]. In South Africa, the National Essential Medicines List Committee uses the WHO MLEM to select and create national EMLs for cancer treatments [98]. The Committee prioritizes procurement of these treatments to minimize drug expenditures [98,100]. China implemented a similar strategy by linking EMLs to policies that finance pharmaceuticals [99]. China’s National Essential Medicines Scheme connects three policies to their National Essential Drug Lists: 1) a ‘zero mark-up policy’ for essential medicines prices; 2) a reimbursement scheme for selected medicines from the National Essential Drugs List, and 3) a requirement for shared local bidding and procurement for essential medicines [98,100]. In both countries, adherence to national EMLs has financial consequences. Compliance results in savings through cost avoidance and encourages bulk or large volume purchases rather than separate smaller purchases from manufacturers. Finally, we note that the MLEM merits continue to be debated [15], in light of the resource implications that are especially intensive in LMICs seeking to align their EMLs with the MLEM [61].

### Devolution of government authority for essential medicines

The devolution of healthcare systems can significantly impact the procurement and distribution of essential medicines. Devolved systems contribute to UHC by enhancing the medicines procurement and distribution process and reducing the reaction time to address healthcare needs. Greater local decision autonomy can reduce bureaucracy and streamline decision-making, allowing local governments to identify areas with the highest need, improving fair distribution of essential medicines, and ultimately resulting in decreases in misallocation, greater accountability, and promoting local capacity building [101]. The modest improvements in UHC experienced by Kenya in the decentralized system were similar to other countries that have implemented devolved systems. In the 1990s, Uganda and the Philippines launched a devolved system to improve access to essential medicines; however, disparities in quality and availability continued to plague various regions in the two countries [102–104]. These mixed results were also reported by other African and Asian countries such as Burkina Faso, Mozambique, Nigeria, Uganda, and Indonesia [105]. Despite increased local autonomy, these countries remain dependent on their central governments’ financial allocations. While devolution benefited larger cities, remote areas faced disadvantages and required additional investment to optimize the benefits of devolution for accessing medicines [105]. Greater support through increased funding or partnerships with counties that have larger budgets could better address these disparities. Policies should incentivize local governments to use joint procurement through shared local bidding or bulk purchases of essential medicines [106]. The savings achieved can support a sustainable and predictable budgeting process by reducing the costs associated with stocking medicines.

### Investment in domestic pharmaceutical manufacturing

The pharmaceutical industry in SSA countries is still emerging, and there remains significant untapped potential for domestic production [107]. In 2020, there were around 600 pharmaceutical manufacturers in Africa, with 80% located in eight countries [108]. The manufacturers are generally small and do not meet international standards for export [107,108]. In contrast, China and India, each with similar populations of approximately 1.4 billion, have 5,000 and 10,500 drug manufacturers, respectively [107]. Lack of investment and limitations on patent and licensing agreements on generic medications play a role in the slow growth of the manufacturing industry in SSA countries [109]. Ethiopia has developed a national action plan, positioning itself as a model for domestic investment in the pharmaceutical industry well before Uganda’s BUBU program. The country is home to nine local manufacturers, and its experience is especially instructive. First, Ethiopia does not impose taxes on medicines but enforces a 5% import tax on finished products [110]. The program has resulted in higher availability of domestically manufactured medicines compared to those imported in the public and private sector [110,111]. Second, the Ethiopian government spent more on local products than on imports, though availability remains a challenge [112]. Local manufacturing also occurs in Kenya and Tanzania, primarily producing *non*-essential medicines [113]. Of the 946 locally produced drugs in Kenya, 310 (21%) were essential medicines, whereas in Tanzania, 39 out of 97 (5%) locally produced medicines are essential medicines [96].

Uganda’s BUBU program demonstrates that investing in domestic production can gradually reduce dependence on external pharmaceutical manufacturers [114]. However, success can only be attained with strong political commitment, effective leadership, and incentives to lead to the transformation of the national pharmaceutical sector [115]. Domestic pharmaceutical production can only succeed if NMPs explicitly outline and implement that commitment. The approach should include monetary and non-monetary incentives for domestic manufacturers. Monetary incentives can include soft loans, while non-monetary incentives include provisions for capacity building and infrastructure development [115]. Building on this strategy, we suggest that designating a preferred domestic manufacturer to produce for the public sector will ensure stable revenue and a sustainable business model for manufacturers. This designation could be awarded on a rotating basis, for a defined period or until a supplier reaches a predefined total revenue or volume. We would also recommend that NMPs provide guidelines for reviewing manufacturer contract performance to minimize product shortages. Creating policies that promote and support robust domestic production of essential medicines with favorable financial terms for manufacturers can significantly reshape the domestic manufacturing landscape in SSA countries.

## Limitations

This scoping review offered a broad overview of essential medicines policies in Kenya and Uganda, two SSA countries representative of the region yet with unique policy developments that are potentially instructive for other SSA countries. Our analysis is not, however, without limitations. First, given the limited literature, we did not critically evaluate the quality of studies included in the review as a separate activity. Instead, we synthesized across the available evidence and accounted for differences in study quality implicitly while building our narrative. Second, our analysis is not exhaustive of all the strategies used by Kenya and Uganda for essential medicines. Our focus on supply chain strategies guided our selection of policies to examine. Third, while we could have selected other high-performing countries in SSA, such as Rwanda or Zimbabwe; our choice to focus on Kenya and Uganda was once again guided by our focus on supply chain reforms in the broader context of essential medicines and UHC policy. Fourth, potential publication bias may exist in the included literature particularly when comparing policies across SSA countries with different health-system structures. However, including the grey literature and government documents in our review should limit the degree to which such bias affects our findings. Finally, we acknowledge the importance of other factors beyond supply chain policies for universal access to essential medicines; however, the policies we review should together improve access to medicines. Establishing their specific causal effects is beyond the scope of this review.

## Conclusions

This review provides an in-depth overview of the key essential medicines supply-chain provisions in Kenya and Uganda, two uniquely instructive SSA countries. Our review identifies strategies that can support a reliable and sustainable path for improving access to essential medicines under UHC in SSA and similar LMICs. Specifically, we recommend: 1) developing robust national EMLs, periodically aligned with the WHO MELM and optimized to reflect local priorities and resource constraints; 2) making EMLs a central guide of NMPs, informing procurement, supply-chain management, financing, and local manufacturing incentives and priorities; and 3) fostering the implementation of a devolved system tailored to the resources available at local and county governments, allowing smaller counties to collectively procure medicines so that no single county bears the burden of high-priced medicines. This should promote geographic and socioeconomic equity in medicines access. We recognize that local devolution may not be fully possible in some countries. However, we assert that local governments and health authorities should be collectively granted a degree of autonomy to periodically determine and update their needed stockpiles of essential medicines under the national EML umbrella, which can then be timely procured and disbursed. These recommendations should inform future research and guide stakeholders in SSA countries to help make essential medicines supply chains more resilient, equitable, and sustainable.

## Supplementary Material

FINAL 6 2submitrev_supplemental tables_Kludze Hussein_essential medicines supply chains Kenya Uganda.docx

## Data Availability

The authors confirm that the data supporting the findings of this study are available within the article’s supplementary materials (Suppl. Table 2)
