# Supplementary material for: Fortifying essential medicines supply chains in Sub-Saharan Africa – the curious cases of Kenya and Uganda
Source: Glob Health Action. 2026 Aug 24;19(1):2718931. doi: 10.1080/16549716.2026.2718931 (PMC13508496; doi:10.1080/16549716.2026.2718931)
Supplement: Supplementary file 1 [file ZGHA_A_2718931_SM3334.docx]

**Supplemental Table 1. Preferred Reporting Items for Systematic reviews and Meta-Analyses extension for Scoping Reviews (PRISMA-ScR) Checklist**

| **SECTION** | **ITEM** | **PRISMA-ScR CHECKLIST ITEM** | **REPORTED ON PAGE #** |
| --- | --- | --- | --- |
| **TITLE** | | | |
| Title | 1 | Identify the report as a scoping review. | 1 |
| **ABSTRACT** | | | |
| Structured summary | 2 | Provide a structured summary that includes (as applicable): background, objectives, eligibility criteria, sources of evidence, charting methods, results, and conclusions that relate to the review questions and objectives. | 2 |
| **INTRODUCTION** | | | |
| Rationale | 3 | Describe the rationale for the review in the context of what is already known. Explain why the review questions/objectives lend themselves to a scoping review approach. | 3-6 |
| Objectives | 4 | Provide an explicit statement of the questions and objectives being addressed with reference to their key elements (e.g., population or participants, concepts, and context) or other relevant key elements used to conceptualize the review questions and/or objectives. | 6 |
| **METHODS** | | | |
| Protocol and registration | 5 | Indicate whether a review protocol exists; state if and where it can be accessed (e.g., a Web address); and if available, provide registration information, including the registration number. | 6-7 |
| Eligibility criteria | 6 | Specify characteristics of the sources of evidence used as eligibility criteria (e.g., years considered, language, and publication status) and provide a rationale. | 7  Table 1 |
| Information sources* | 7 | Describe all information sources in the search (e.g., databases with dates of coverage and contact with authors to identify additional sources), as well as the date the most recent search was executed. | 7 |
| Search | 8 | Present the full electronic search strategy for at least 1 database, including any limits used, such that it could be repeated. | 7-9 |
| Selection of sources of evidence† | 9 | State the process for selecting sources of evidence (i.e., screening and eligibility) included in the scoping review. | 7 |
| Data charting process‡ | 10 | Describe the methods of charting data from the included sources of evidence (e.g., calibrated forms or forms that have been tested by the team before their use, and whether data charting was done independently or in duplicate) and any processes for obtaining and confirming data from investigators. | 9  Table 2 |
| Data items | 11 | List and define all variables for which data were sought and any assumptions and simplifications made. | N/A |
| Critical appraisal of individual sources of evidence§ | 12 | If done, provide a rationale for conducting a critical appraisal of included sources of evidence; describe the methods used and how this information was used in any data synthesis (if appropriate). | Incorporated in narrative synthesis |
| Synthesis of results | 13 | Describe the methods of handling and summarizing the data that were charted. | 9 |
| **RESULTS** | | | |
| Selection of sources of evidence | 14 | Give numbers of sources of evidence screened, assessed for eligibility, and included in the review, with reasons for exclusions at each stage, ideally using a flow diagram. | 9 |
| Characteristics of sources of evidence | 15 | For each source of evidence, present characteristics for which data were charted and provide the citations. | 9 |
| Critical appraisal within sources of evidence | 16 | If done, present data on critical appraisal of included sources of evidence (see item 12). | Incorporated in narrative synthesis |
| Results of individual sources of evidence | 17 | For each included source of evidence, present the relevant data that were charted that relate to the review questions and objectives. | 9 |
| Synthesis of results | 18 | Summarize and/or present the charting results as they relate to the review questions and objectives. | Supplemental Table 2 |
| **DISCUSSION** | | | |
| Summary of evidence | 19 | Summarize the main results (including an overview of concepts, themes, and types of evidence available), link to the review questions and objectives, and consider the relevance to key groups. | 21-26 |
| Limitations | 20 | Discuss the limitations of the scoping review process. | 26-27 |
| Conclusions | 21 | Provide a general interpretation of the results with respect to the review questions and objectives, as well as potential implications and/or next steps. | 27-28 |
| **FUNDING** | | | |
| Funding | 22 | Describe sources of funding for the included sources of evidence, as well as sources of funding for the scoping review. Describe the role of the funders of the scoping review. | 29 |

*From:* Tricco AC, Lillie E, Zarin W, O'Brien KK, Colquhoun H, Levac D, et al. PRISMA Extension for Scoping Reviews (PRISMAScR): Checklist and Explanation. Ann Intern Med. 2018;169:467–473. [doi: 10.7326/M18-0850](http://annals.org/aim/fullarticle/2700389/prisma-extension-scoping-reviews-prisma-scr-checklist-explanation).

**Supplemental Table 2. Included Studies**

| Authors | Publication Year | Title | Objective | Publication |
| --- | --- | --- | --- | --- |
| Leung NH, Chen A, Yadav P, Gallien J. | 2016 | The Impact of Inventory Management on Stock-Outs of Essential Drugs in Sub-Saharan Africa: Secondary Analysis of a Field Experiment in Zambia | inventory control policies | PLoS One. 2016 May 26;11(5):e0156026. doi: 10.1371/journal.pone.0156026. eCollection 2016. |
| Manji I, Manyara SM, Jakait B, Ogallo W, Hagedorn IC, Lukas S, Kosgei EJ, PastakiaSD. | 2016 | The Revolving Fund Pharmacy Model: backing up the Ministry of Health supply chain in western Kenya | evolving Fund Pharmacy Model | Int J Pharm Pract. 2016 Oct;24(5):358-66. doi: 10.1111/ijpp.12254. Epub2016 Feb 23. |
| Trap B, Kikule K, Vialle-Valentin C, Musoke R, Lajul GO, Hoppenworth K, Konradsen D. | 2016 | First regulatory inspections measuring adherence to Good Pharmacy Practices in the public sector in Uganda: a cross-sectional comparison of performance between supervised and unsupervised facilities | medicines management capacity (GPP and SPARS) | J Pharm Policy Pract. 2016 May 4;9:18. doi: 10.1186/s40545-016-0068-4. eCollection 2016. |
| Ndomondo-Sigonda, Margareth; Miot, Jacqueline; Naidoo, Shan; Dodoo, Alexander; Kaale, Eliangiringa; | 2017 | Medicines regulation in Africa: current state and opportunities | summary of the current status of National Medicines Regulatory Authorities (NMRAs) in Africa | Pharmaceutical medicine 2017;31(6):383 |
| Suleman, Fatima; Gray, Andy; | 2017 | Pharmaceutical policy in South Africa | medicine policies in the post-apartheid era in South-Africa | Pharmaceutical policy in countries with developing healthcare systems, Springer, p285-302 |
| Magadzire BP, Marchal B, Mathys T, Laing RO, Ward K. | 2017 | Analyzing implementation dynamics using theory-driven evaluation principles: lessons learnt from a South African centralized chronic dispensing model | centralized dispensing of essential medicines | BMC Health Serv Res. 2017 Dec 4;17(Suppl 2):724. doi: 10.1186/s12913-017-2640-2. |
| Linnander E, Yuan CT, Ahmed S, Cherlin E, Talbert-Slagle K, Curry LA. | 2017 | Process evaluation of knowledge transfer across industries: Leveraging Coca-Cola's supply chain expertise for medicine availability in Tanzania | partnership designed to improve the availability of medical supplies | PLoS One. 2017 Nov 9;12(11):e0186832. doi: 10.1371/journal.pone.0186832. eCollection 2017. |
| Vian T, Kohler JC, Forte G, Dimancesco D. | 2017 | Promoting transparency, accountability, and access through a multi-stakeholder initiative: lessons from the medicines transparency alliance | promoting access goals through greater transparency | J Pharm Policy Pract. 2017 Jun 2;10:18. doi: 10.1186/s40545-017-0106-x. eCollection 2017. |
| Wirtz VJ, Hogerzeil HV, Gray AL, Bigdeli M, de Joncheere CP, Ewen MA, Gyansa-LutterodtM, Jing S, Luiza VL, Mbindyo RM, Möller H, Moucheraud C, PécoulB, Rägo L, Rashidian A, Ross-Degnan D, Stephens PN, Teerawattananon Y, 't Hoen EF, Wagner AK, Yadav P, Reich MR. | 2017 | Essential medicines for universal health coverage |  | Lancet. 2017 Jan 28;389(10067):403-476. |
| Pastakia, Sonak; Njuguna, Benson; Tran, Dan N; | 2018 | Problems and obstacles of poorest countries in having good governance and quality and effective pharmaceutical policy | corruption, lack of implementation framework, and implementation gaps present as barriers to effective policies and good governance in LMIC | Social and Administrative Aspects of Pharmacy in Low-and Middle-Income Countries, Elsevier, p387-401 |
| Martei YM, Chiyapo S, Grover S, Ramogola-Masire D, Dryden-Peterson S, Shulman LN, Tapela N. | 2018 | Availability of WHO Essential Medicines for Cancer Treatment in Botswana | Botswana National Essential Medicines List (NEML) for cancer drugs with the WHO's Essential Medicines List (EML) | J Glob Oncol. 2018 Sep;4:1-8. doi: 10.1200/JGO.17.00063. |
| Briggs J, Embrey M, Maliqi B, Hedman L, Requejo J. | 2018 | How to assure access of essential RMNCH medicines by looking at policy and systems factors: an analysis of countdown to 2015 countries | highlights country deficiencies in policies and systems, such as incoherent policy guidelines, problems in product registration, lack of logistics data, and central-level stock-outs | BMC Health Serv Res. 2018 Dec 7;18(1):952. doi: 10.1186/s12913-018-3766-6. |
| Armstrong-Hough M, Kishore SP, Byakika S, Mutungi G, Nunez-Smith M, Schwartz JI. | 2018 | Disparities in availability of essential medicines to treat non-communicable diseases in Uganda: A Poisson analysis using the Service Availability and Readiness Assessment |  | PLoS One. 2018 Feb 8;13(2):e0192332. doi: 10.1371/journal.pone.0192332. eCollection 2018. |
| Mackintosh M, Tibandebage P, Karimi Njeru M, Kariuki Kungu J, Israel C, MujinjaPGM. | 2018 | Rethinking health sector procurement as developmental linkages in East Africa | describe the challenges faced in accessing NCD medicines in Kenya during the pandemic | Soc Sci Med. 2018 Mar;200:182-189. doi: 10.1016/j.socscimed.2018.01.008. Epub 2018 Jan 31. |
| Oteba M, Wagner AK, Seru M, Embrey M, Trap B. | 2018 | Pharmaceutical system strengthening in Uganda: implementing a holistic, evidence-informed, long-term strategy | planning for strengthening access to cancer medicines for children: pooled procurement, evidence-based forecasting, and regional harmonization of regulatory processes | J Pharm Policy Pract. 2018 Oct 15;11:23. doi: 10.1186/s40545-018-0150-1. eCollection 2018. |
| Bizimana T, Kayumba PC, Heide L. | 2020 | Prices, availability and affordability of medicines in Rwanda | medicine prices, affordability, availability | PLoS One. 2020 Aug 3;15(8):e0236411. doi: 10.1371/journal.pone.0236411. PMID: 32745100; PMCID: PMC7398547. |
| Tran DN, Manji I, Njuguna B, Kamano J, Laktabai J, Tonui E, Vedanthan R, PastakiaS. | 2020 | Solving the problem of access to cardiovascular medicines: revolving fund pharmacy models in rural western Kenya | revolving fund pharmacies (RFPs) | BMJ Glob Health. 2020 Nov;5(11):e003116. doi: 10.1136/bmjgh-2020-003116. |
| Tirivangani T, Alpo B, Kibuule D, Gaeseb J, Adenuga BA. | 2021 | Impact of COVID-19 pandemic on pharmaceutical systems and supply chain - a phenomenological study | inequitable access to essential medicines in the public and private sectors of Namibia | Explor Res Clin Soc Pharm. 2021 Jun;2:100037. doi: 10.1016/j.rcsop.2021.100037. Epub 2021 Jun 24. |
| Alemu T, Jemal A, Gashe F, Suleman S, Sudhakar S, Fekadu G. | 2021 | Integrated pharmaceutical logistics system implementation in selected health facilities of Ethiopia: The case of four WOLLEGA ZONES | indicators in the availability of essential health commodities since Integrated Pharmaceuticals Logistics System (IPLS) | Res Social Adm Pharm. 2021 May;17(5):956-968. doi: 10.1016/j.sapharm.2020.07.026. Epub 2020 Aug 6. |
| Chen J, Ssennyonjo A, Wabwire-Mangen F, Kim JH, Bell G, Hirschhorn L. | 2021 | Does decentralization of health systems translate into decentralization of authority? A decision space analysis of Ugandan healthcare facilities | Facility-level autonomy in drug procurement | Health Policy Plan. 2021 Oct 12;36(9):1408-1417. doi: 10.1093/heapol/czab074. |
| Mhazo, Alison T; Maponga, Charles C; | 2022 | Framing access to essential medicines in the context of Universal Health Coverage: a critical analysis of health sector strategic plans from eight countries in the WHO African region | Access to essential medicines in the context of Universal Health Coverage | BMC Health Services Research 2025;22(1):1390. |
| Toroitich AM, Dunford L, Armitage R, Tanna S. | 2022 | Patients Access to Medicines - A Critical Review of the Healthcare System in Kenya | review of health care system and supply chain structure | Risk Manag Healthc Policy. 2022 Mar 1;15:361-374. doi: 10.2147/RMHP.S348816. eCollection 2022. |
| Lugada E, Komakech H, Ochola I, Mwebaze S, Olowo Oteba M, Okidi Ladwar D. | 2022 | Health supply chain system in Uganda: current issues, structure, performance, and implications for systems strengthening | structure, performance, and challenges of the health supply chain system in Uganda | J Pharm Policy Pract. 2022 Mar 1;15(1):14. doi: 10.1186/s40545-022-00412-4. |
| Lugada E, Ochola I, Kirunda A, SembatyaM, Mwebaze S, Olowo M, Ladwar DO, Komakech H. | 2022 | Health supply chain system in Uganda: assessment of status and of performance of health facilities | status and performance of the supply chain system across all levels of health facilities in Uganda | J Pharm Policy Pract. 2022 Oct 5;15(1):58. doi: 10.1186/s40545-022-00452-w. |
| Muyingo S, Etoori D, Lotay P, Malamba S, Olweny J, Keesler K, Wiersma S, Jjemba P, Settaala R. | 2022 | The procurement and supply chain strengthening project: improving public health supply chains for better access to HIV medicines, Uganda 2011-2016 | procurement and supply chain strengthening project in Uganda | J Pharm Policy Pract. 2022 Oct 27;15(1):72. doi: 10.1186/s40545-022-00467-3. |
| Kamba PF, NambatyaW, Aguma HB, Charani E, Rajab K. | 2022 | Gaps and opportunities in sustainable medicines use in resource limited settings: A situational analysis of Uganda | Uganda’s challenges in providing sustainable medicines despite adoption of a national medicines policy, essential medicines list, a medicines regulation framework and promotion of domestic production | Br J Clin Pharmacol. 2022 Sep;88(9):3936-3942. doi: 10.1111/bcp.15324. Epub2022 Apr 8. |
| Petricca K, Kambugu J, Githang'a J, Macharia WM, Njuguna F, McLigeyo A, NyangasiM, Orem J, Kanyamuhunga A, Laiti R, Katabalo D, Schroeder K, Rogo K, Maguire B, Wambui L, Nkurunziza JN, Wong B, Neposlan J, KilaweL, Gupta S, Denburg AE. | 2023 | Access to essential cancer medicines for children: a comparative mixed-methods analysis of availability, price, and health-system determinants in east Africa | Recurrent stockouts of a wide range of cytotoxic and supportive care medicines | Lancet Oncol. 2023 May;24(5):563-576. doi: 10.1016/S1470-2045(23)00072-4. Epub2023 Apr 3. |
| Ipagala P, Mlugu EM, Mwakalukwa R, Kagashe GA. | 2023 | Impact of COVID-19 on the supply chain of essential health commodities: a mixed method study, in Dar es Salaam, Tanzania | Importers of essential health commodities and local pharmaceutical manufacturers | J Pharm Policy Pract. 2023 Sep 22;16(1):103. doi: 10.1186/s40545-023-00617-1. |
| Yemeke TT, Umaru FA, Ferrand RA, Ozawa S. | 2023 | Impact of the COVID-19 Pandemic on Medical Product Procurement, Prices, and Supply Chain in Zimbabwe: Lessons for Supply Chain Resiliency | Pooled procurement, especially at regional and global levels, with long-term service agreements | Glob Health Sci Pract. 2023 Oct 30;11(5):e2200424. doi: 10.9745/GHSP-D-22-00424. Print 2023 Oct 30. |
| Hailu R, Gizaw T, Berhanu N, Mulugeta T, Boche B, Gudeta T. | 2023 | Exploring the role of ICT in pharmaceutical supply chain practices and operational performance in Ethiopia: a structural equation modeling approach | Information Communication Technology (ICT) is one of the strategies for optimizing supply chain coordination | BMC Health Serv Res. 2023 Jun 14;23(1):634. doi: 10.1186/s12913-023-09627-w. |
| Mabizela S, NakambaleHN, Bangalee V. | 2023 | Predictors of medicine redistribution at public healthcare facilities in King Cetshwayo District, KwaZulu-Natal, South Africa | inventory management, particularly regarding stockouts, overstocking, and expiry-related wastage | BMC Health Serv Res. 2023 Oct 17;23(1):1108. doi: 10.1186/s12913-023-10096-4. |
| Umer A, Mohammed H, Yazie B, Angaw DA, Gonete TZ, EndehabtuBF, Tilahun B, Jisso M, Tamiso A, Assesfa NA, Alemayehu A, Fikre R, Mizana BA, Dessie K, Sime H, Abera M, Mecha M, Yesuf EA, Gurmu KK, Kebede M. | 2023 | Assessment of Availability of Tracer Drugs and Basic Diagnostics at Public Primary Health Care Facilities in Ethiopia During COVID-19 Pandemic | mean availability of tracer drugs | Ethiop J Health Sci. 2023 Oct;33(Spec Iss 2):135-142. doi: 10.4314/ejhs.v33i2.7S. |
| Kamere N, Rutter V, Munkombwe D, AywakDA, Muro EP, Kaminyoghe F, Rajab K, Lawal MO, Muriithi N, Kusu N, Karimu O, Barlatt SHA, NambatyaW, Ashiru-Oredope D. | 2023 | Supply-chain factors and antimicrobial stewardship (why didn't it appear in Uganda) | focus on a specific disease state or condition - antimicrobial stewardship | Bull World Health Organ. 2023 Jun 1;101(6):403-411. doi: 10.2471/BLT.22.288650. Epub 2023 Apr 17. |
| Kyalisiima I, Kitutu FE, Gibson L, Akaso I, Ndaabe A, Aguma HB, Musoke D, Adome RO, Kutyabami P. | 2023 | Compliance of public health facilities with essential medicines and health supplies redistribution guidelines in Mbale district, Eastern Uganda: a mixed-methods study | Redistribution of essential medicines and health supplies strategy toaddress supply chain uncertainty | J Pharm Policy Pract. 2023 Feb 28;16(1):32. doi: 10.1186/s40545-023-00545-0. |
| Ravinetto, Raffaella; Henriquez, Rodrigo; Srinivas, Prashant N; Bradley, Hazel; Coetzee, Renier; Ochoa, Theresa J; Ngabonziza, Jean Claude Semuto; Mazarati, Jean-Baptiste; Van Damme, Wim; van de Pas, Remco | 2024 | Shaping the future of global access to safe, effective, appropriate and quality health products | Inequitable access to essential health products during the COVID-19 pandemic | BMJ Global Health 2024;9(1):e014425 |
| Kabera JC, Mukanyangezi MF. | 2024 | Influence of inventory management practices on the availability of emergency obstetric drugs in Rwandan public hospitals: a case of Rwanda Southern Province | Adequate supply chain staffing in health facilities is the most important key to improving inventory management practices and medicine availability | BMC Health Serv Res. 2024 Jan 4;24(1):14. doi: 10.1186/s12913-023-10459-x. |
| Mohan S, Mangal TD, Colbourn T, Chalkley M, Chimwaza C, Collins JH, Graham MM, Janoušková E, Jewell B, Kadewere G, Li Lin I, Manthalu G, Mfutso-Bengo J, Mnjowe E, Molaro M, Nkhoma D, Revill P, She B, Manning Smith R, Tafesse W, Tamuri AU, Twea P, Phillips AN, Hallett TB. | 2024 | Factors associated with medical consumable availability in level 1 facilities in Malawi: a secondary analysis of a facility census | Product availability; private-for-profit ownership had 63% (1·63, 1·40-1·89) and 49% (1·49, 1·24-1·80) higher odds respectively than government-owned facilities | Lancet Glob Health. 2024 Jun;12(6):e1027-e1037. doi: 10.1016/S2214-109X(24)00095-0. |
| Onyango JO, Ogira D, Kokwaro G. | 2024 | Availability of essential medicines during the COVID-19 pandemic: A qualitative study examining experiences and level of preparedness in Kenya | regulatory policy weaknesses such as an inadequate legal framework | PLOS Glob Public Health. 2024 Jul 29;4(7):e0002547. doi: 10.1371/journal.pgph.0002547. eCollection 2024. |
| Koduah A, Kusu N, Ahimon H, Brown A, Njeru N, Menge T, Ouattara M, Upadhyay N, Upadhyaya MK, Jiha M, Hodgson R, Nfor E, Aboagye-Nyame F, Hafner T. | 2024 | An exploratory study of the mandate and functions of national pharmaceutical services units: global trends and the cases of Côte d'Ivoire, Kenya, and Nepal | pharmaceutical care and policy oversight | J Pharm Policy Pract. 2024 Dec 13;17(1):2430438. doi: 10.1080/20523211.2024.2430438. eCollection 2024. |
| Petricca K, Carson L, Kambugu J, Denburg A. | 2024 | Strengthening access to cancer medicines for children in East Africa: policy options to enhance medicine procurement, forecasting, and regulations | Policy options for pooled procurement, evidence-based forecasting, and regional harmonization of regulatory processes for cancer medications | Glob Health Res Policy. 2024 Jul 1;9(1):24. doi: 10.1186/s41256-024-00365-y. |
| Nambahu, Fransina Netumbo; | 2025 | Situation analysis of the medicines pricing in context of the national medicines policy of Namibia | National medicine policy pricing | University of the Western Cape |
| Otaigbe, Idemudia Imonikhe; | 2025 | Mitigating inequitable access to appropriate antibiotics in low-and middle-income countries | discusses factors responsible for inequitable access to appropriate antibiotics in LMICs | JAC-Antimicrobial Resistance 2025;7(2):dlaf061 |
| Mekonen ZT, Cho DJ, Fenta TG. | 2025 | Health commodities logisticsmanagement information system performance at public health facilities of Amhara region, Ethiopia | Logistics Management Information Systems - unreliable infrastructure, poor internet connectivity, and insufficient human resources impede effective LMIS performance. | Front Public Health. 2025 Mar 24;13:1545429. doi: 10.3389/fpubh.2025.1545429. eCollection 2025. |
| Kenya | 2010 | HIV and AIDS Prevention and Control Act: PART IV – TESTING, SCREENING AND ACCESS TO HEALTHCARE SERVICE | The Government shall, to the maximum of its available resources, take the steps necessary to ensure the access to essential healthcare services, including the access to essential medicines at affordable prices by persons with HIV or AIDS and those exposed to the risk of HIV infection. | Government document <https://nsdcc.go.ke/wp-content/uploads/2022/07/HIVandAIDSPreventionandControlAct14of2006.pdf> |
| Kenya | 2022 | Kenya Medical Supplies Authority Act: PART IV – MISCELLANEOUS PROVISIONS | The Kenya Medical Supplies Authority Act “shall put into place measures to ensure maintenance and sustenance of strategic reserves of essential medicines and medical supplies” | Government document <https://new.kenyalaw.org/akn/ke/act/2013/20/eng@2022-12-31> |
| Kenya | 2014 | Kenya Health Policy 2014-2030 | National policy whose goal is to attain the highest possible standard of health in a responsive manner. | Government document <https://arua-ncd.org/wp-content/uploads/2022/10/kenya-health-policy.pdf> |
| Kenya | 2017 | Health Act | Right to health including essential medicines; The national government shall provide guidelines for the procurement, distribution and management of health products and technologies including essential medicines, laboratory chemicals and reagents and non-pharmaceuticals at all levels of the national health system. | Government document <https://new.kenyalaw.org/akn/ke/act/2017/21/eng@2022-12-31> |
| Kenya | 2020 | Kenya Universal Health Coverage Policy 2020 – 2030 | Aims to provide all citizens with access to quality essential health services without facing financial hardshipincluding essential medicines | Government document <http://guidelines.health.go.ke:8000/media/Kenya_Universal_Health_Coverage_Policy_2020__2030.pdf> |
| Kenya | 2020 | Health Products and Technologies Supply Chain Strategy 2020-2025 | Seeks to improve coordination, provide direction, decision-making, and investments to provide consistent health products and technologies | Government document <http://guidelines.health.go.ke:8000/media/Health_Products_and_Technogies_Supply_Chain_Strategy-_2020-2025.pdf> |
| Kenya | 2022 | The Kenya Constitution | Article 43.(1a) provides that “every person has the right to the highest attainable standard of health, which includes the right to healthcare services” | Government document <https://www.parliament.go.ke/sites/default/files/2017-05/The_Constitution_of_Kenya_2010.pdf> |
| Kenya | 2023 | Kenya Essential Medicines List | A list that ensures that medicines required at all healthcare levels are included to guide efforts to improve access, in line with achieving UHC | Government document <http://guidelines.health.go.ke:8000/media/Kenya_Essential_Medicines_List_2023_qbkhTlV.pdf> |
| Uganda | 2010 | The National Health Policy II | "Facilitate access of the private sector access to development capital, essential medicines and supplies for healthcare developments vital to service expansion to the population" | Government document <https://library.health.go.ug/sites/default/files/resources/Second%20National%20Health%20Policy%202010.pdf> |
| Uganda | 2012 | Uganda National Redistribution Strategy for Prevention of Expiry and Handling of Expired Medicines and Health Supplies | address the challenges of medicines and Health supplies management at the facility level. | Government document <https://library.health.go.ug/medical-products-technologies/medicines/uganda-national-redistribution-strategy-prevention-expiry> |
| Uganda | 2014 | Buy Uganda Build Uganda policy | "...promote the consumption/usage of locally originating products" | Government document  <https://www.mtic.go.ug/bubu-certification-mark/> |
| Uganda | 2015 | National Medicines Policy | "...ensuring access to, and appropriate use of good quality, affordable essential medicines, and health supplies" | Government document  <https://www.health.go.ug/wp-content/uploads/2019/11/National-Medicines-Policy-2015-6.pdf>. |
| Uganda | 2017 | Supervision, Performance Assessment and Recognition Strategy (SPARS) Implementation Guidelines | national strategy to build district level capacity in supply chain | Government document <https://library.health.go.ug/medical-products-technologies/medicines/supervision-performance-assessment-and-recognition-strategy> |
| Uganda | 1993 | National Drug Policy and Authority Statute | "Mandate: to ensure the availability, at all times, of essential, efficacious and cost-effective drugs to the entire population of Uganda as a means of providing satisfactory healthcare and safeguarding the appropriate use of drugs"; '"Promote local production of essential drugs" | Government document  <https://www.nda.or.ug/nda-profile-page/> |
| Uganda | 2021 | 10-Year Health Supply Chain Roadmap for the period 2021/22 to 2031/32 | "The Roadmap seeks to strengthen the capacity of the Government of Uganda’s ministries, departments, and agencies (MDAs) to plan, finance, and effectively manage the national health commodities supply chain priority areas independent of donor support" | Government document <https://library.health.go.ug/medical-products-technologies/medicines/10-year-roadmap-government-ugandas-health-supply-chain-self> |
| Uganda | 2023 | Uganda Essential Medicines and Health Supplies List for Uganda (EMHSLU) 2023 Publication | "The Essential Medicines and Health Supplies List for Uganda (EMHSLU) 2023 reflects the policy of the Ministry of Health, for the appropriate procurement of safe and efficacious essential medicines, health and laboratory supplies in public institutions" | Government document <https://library.health.go.ug/medical-products-technologies/pharmaceuticals-and-drugs/essential-medicines-and-health-supplies> |
